# Follicular cholangitis mimicking a common bile duct cancer: a case report

**DOI:** 10.1186/s40792-023-01708-6

**Published:** 2023-07-05

**Authors:** Kenji Koneri, Takanori Goi, Hokahiro Katayama, Noriyuki Tagai, Michiaki Shimada, Hidetaka Kurebayashi, Katsuji Sawai, Mitsuhiro Morikawa, Masato Tamaki, Yasuo Hirono, Satomi Hatta, Yoshiaki Imamura, Makoto Murakami

**Affiliations:** 1 First Department of Surgery, University of Fukui, Fukui, 9101193 Japan; 2 Department of Surgery, Tsukushino Hospital, Fukui, 9100102 Japan; 3 Cancer Care Portion Center, University of Fukui, Fukui, 9101193 Japan; 4 Division of Diagnostic Pathology/Surgical Pathology, University of Fukui, Fukui, 9101193 Japan

**Keywords:** Follicular cholangitis, Distal common bile duct, Common bile duct cancer, Case report

## Abstract

**Background:**

Follicular cholangitis (FC) is a benign bile duct disease that was first reported 2003. Pathologically, it is characterized by lymphoplasmacytic infiltration with multiple lymphoid follicle formations under the mucosal layer of the biliary tract. However, as this disease is extremely rare, little is known about its etiology and pathogenesis.

**Case presentation:**

A 77-year-old woman was diagnosed with middle bile duct stenosis and potential increases in alkaline phosphatase (ALP) and γ-glutamyl transpeptidase levels (γ-GTP). Carcinoembryonic antigen (CEA), carbohydrate antigen 19-9 (CA19-9) and IgG4 levels were all within the normal limits. Contrast-enhanced computed tomography (CE-CT) and magnetic resonance imaging (MRI) revealed bile duct dilation from intrahepatic to upper common bile duct and an irregular mass lesion in distal bile duct. Additionally, multiple overlapping leaf-like folds were detected. ^18^F-fluorodeoxyglucose positron emission tomography–computed tomography (^18^F-FDG-PET/CT) did not demonstrate fluorodeoxyglucose uptake. Subtotal stomach-preserving pancreaticoduodenectomy with regional lymph node dissection was performed because common bile duct cancer could not be ruled out. The resected specimen showed diffuse homogeneous middle bile duct wall thickening. Microscopically, the lesion exhibited thick fibrosis with several invaded lymphoplasmacytic cells, and lymphoid follicle formations were detected under the mucosal layer. Immunohistochemical staining (IHC) revealed positive for CD3, CD4, CD20 and CD79a, and these findings led to a final diagnosis of FC. The patient has not experienced recurrence to date (42 months postoperatively).

**Conclusions:**

Currently, accurate preoperative diagnosis of FC is difficult. More cases must be accumulated to generate additional knowledge on its precise diagnosis and proper treatment.

## Background

Several cases of benign biliary tract lesions have been reported previously, and some of these cases have been studied and described well. However, there are limited reports of FC because of its rarity and since neither surgeons nor pathologists are adequately equipped to recognize it [1, 2]. Its etiology remains unknown, and it grossly presents as localized thickening of the bile duct and lymphoplasmacytic cell invasion with many lymphoid follicle formations under the mucosal layer. Some IHC findings, such as CD3, CD4, CD8, CD20 and CD79a positivity, can differentiate FC from other benign biliary disease [3]. We report a case of FC that was difficult to differentiate from distal cholangiocarcinoma, with review of literature.

## Case presentation

A 77-year-old woman who had been prescribed medical treatment for hypertension by a local physician was referred to our hospital because of elevated serum alkaline ALP and γ-GTP levels. She did not complain of any symptoms and had no history of biliary tract disease, abdominal surgery, autoimmune disease, allergies, or abdominal trauma. She also had no history of heavy alcohol consumption or smoking. Her brother had a history of pancreatic cancer. Neither skin yellowing nor abdominal tumor was observed. The patient’s general condition was good, and she had a normal body mass index (22.7 kg/m^2^).

Laboratory examination results revealed slightly elevated serum aspartate aminotransferase (52 IU/L; reference: 13–30 IU/L), alanine aminotransferase (54 IU/L; reference: 17–23 IU/L), ALP (414 IU/L; reference: 106–322 IU/L), and γ-GTP (449 IU/L; reference: 9–32 IU/L) levels. Neither serum total bilirubin nor direct bilirubin was elevated. The patient was positive for serum hepatitis C virus antibody, and serum tumor markers (CEA, CA19-9) and IgG4 levels were within the normal limits.

We performed upper gastrointestinal endoscopy and a total colonoscopy preoperatively to rule out inflammatory bowel disease or other diseases, neither of which revealed any lesions.

**Fig. 1:**
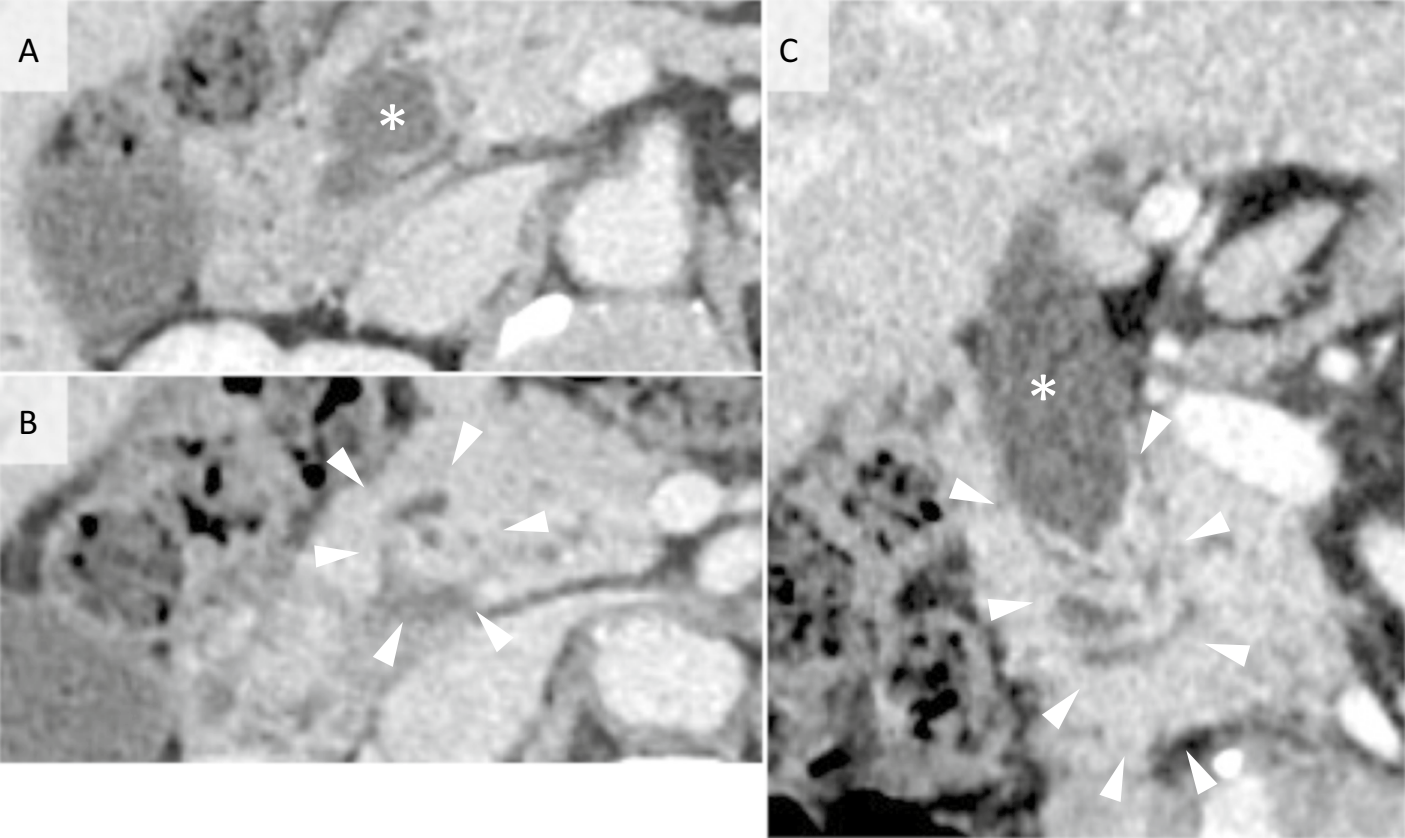
The preoperative contrast-enhanced CT scan. **A** Axial view showing dilated proximal side of common bile duct (asterisk). **B** An irregular mass lesion in middle bile duct (arrowheads). **C** Coronal view showing dilated proximal bile duct (asterisk) and multiple overlapping leaf-like folds in the middle bile duct (arrowheads)

**Fig. 2:**
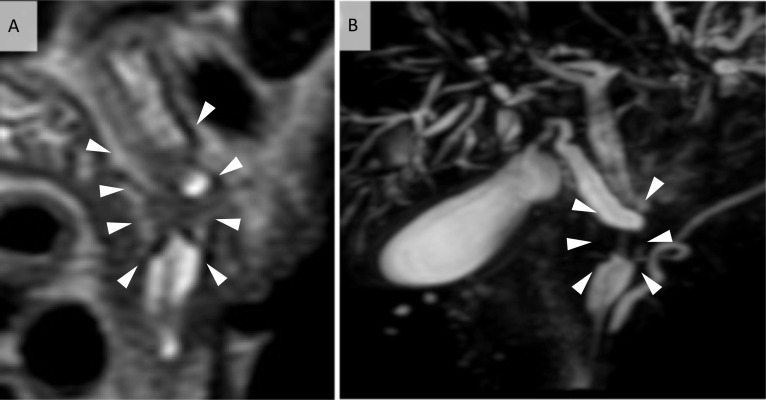
MRI. **A** Coronal section of T2-weighted imaging showing an irregular signal defect in the middle bile duct (arrowheads). **B** Magnetic resonance cholangiopancreatography showing mild dilation of proximal bile duct (arrowheads)

Axial view of CE-CT showed dilation from intrahepatic to upper common bile duct (Fig. 1a), and an irregular mass lesion in distal bile duct (Fig. 1b). On the coronal view, this lesion appeared as multiple overlapping leaf-like folds (Fig. 1c). T2-weighted MRI showed irregular signal defect in the middle bile duct (Fig. 2a). Magnetic resonance cholangiopancreatography (MRCP) also indicated a signal defect and mild dilation of the proximal bile duct (Fig. 2b). ^18^F-FDG-PET/CT revealed no uptake in this lesion. Both biliary cytology and brushing cytology were performed twice, but no malignant disease was proven in all specimens.

Since distal bile duct cancer could not be completely ruled out in this case, we decided to perform surgical treatment with full patient consent. Subtotal stomach-preserving pancreaticoduodenectomy with regional lymph node dissection was performed. The patient had a good clinical course and was discharged on the 35th day postoperatively.

**Fig. 3:**
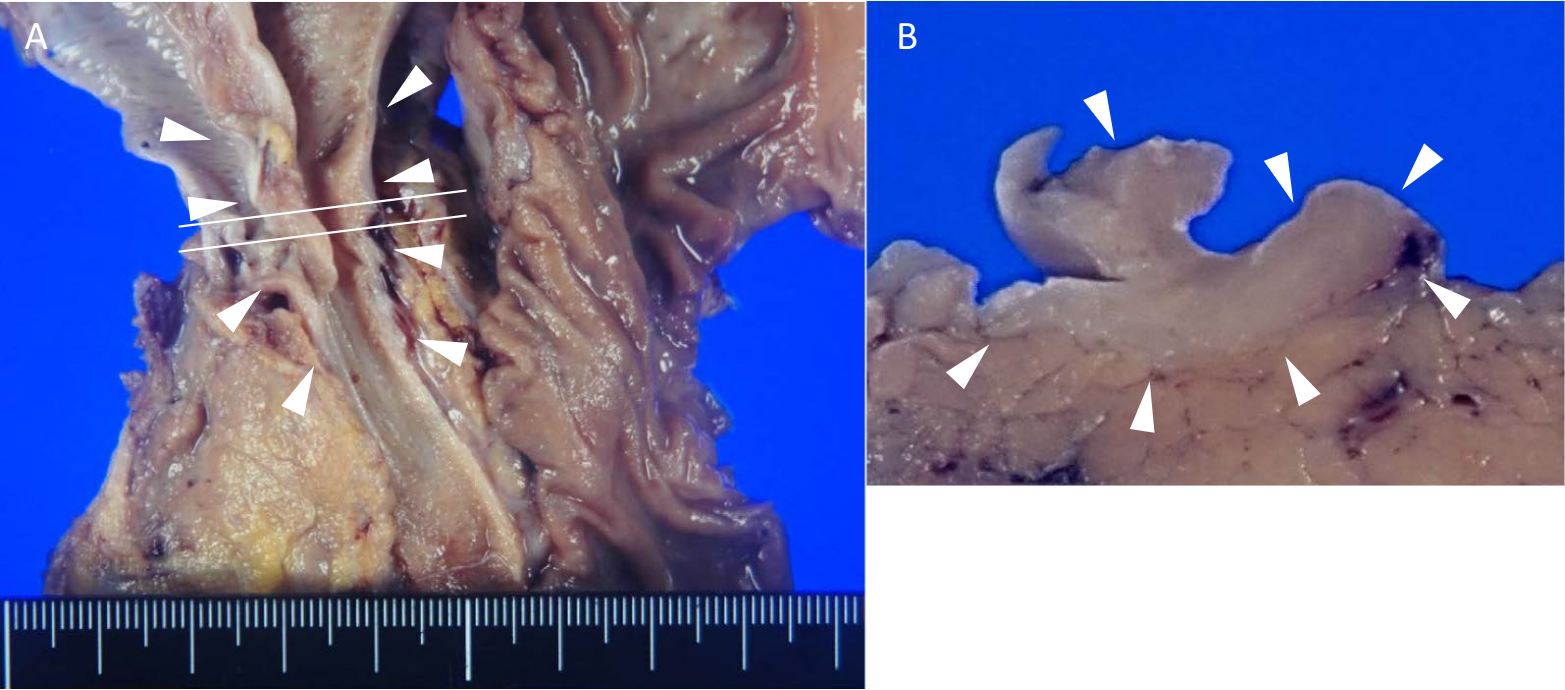
Macroscopic findings. **A** The resected specimen exhibits the middle bile duct wall thickening (arrowheads). **B** Cross section showing diffuse homogenous bile duct wall thickening (arrowheads)

**Fig. 4:**
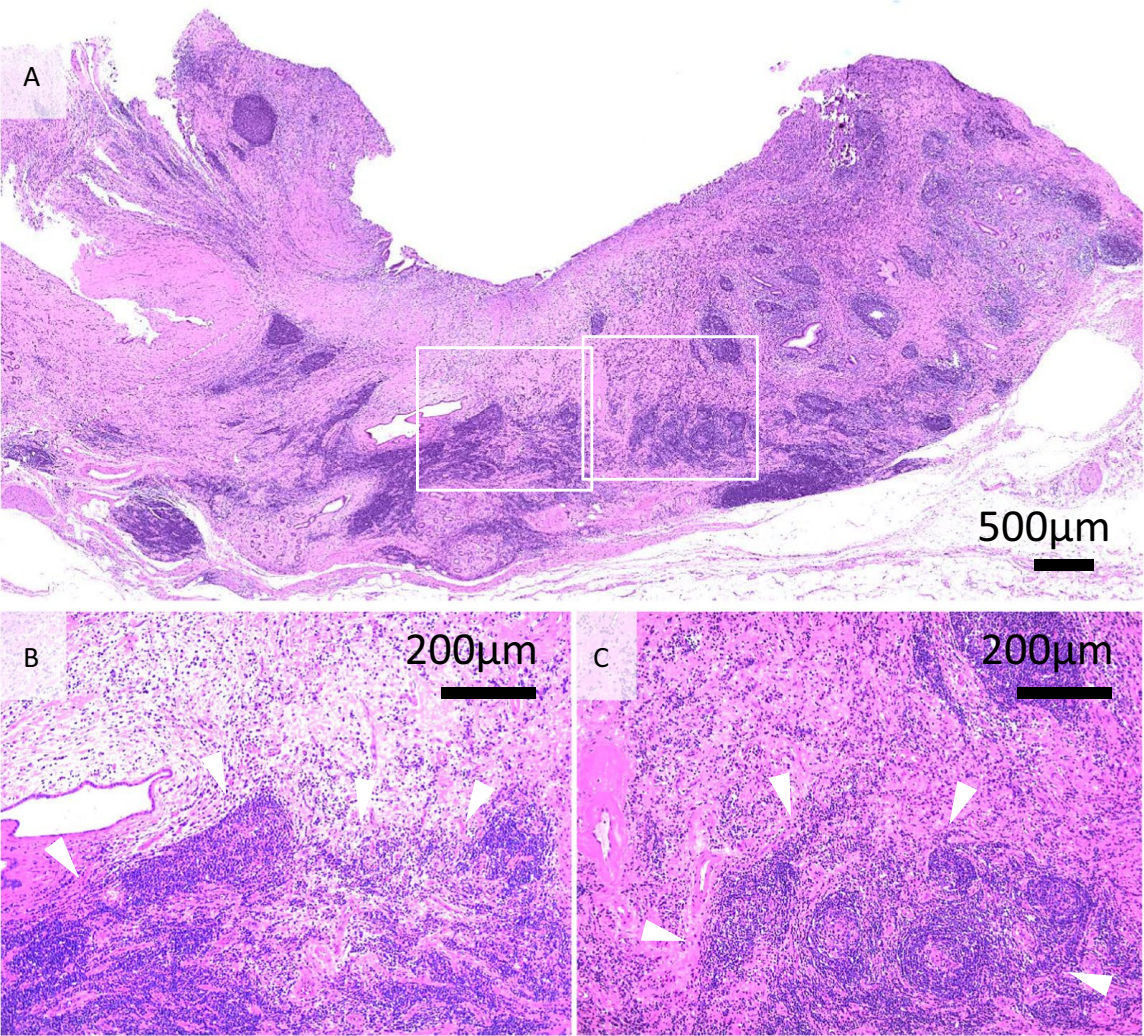
Microscopic findings (hematoxylin & eosin). **A** The bile duct lesion exhibits dense fibrosis under the mucosal layer and severe inflammation of the peribiliary glands. **B** Marked lymphoplasmacytic cells invasion (arrowheads) is observed under the dense fibrosis. **c** Lymphoplasmacytic cells formed numerous lymphoid follicles (arrowheads) containing germinal centers

The resected specimen exhibited bile duct wall thickening in the middle bile duct (Fig. 3a). Cut surface showed diffuse homogenous bile duct wall thickening, but no evidence of invasion into the pancreas (Fig. 3b). Microscopically, the bile duct lesion showed dense fibrosis under the mucosal layer and severe inflammation of the peribiliary glands (Fig. 4a). In the magnified view, marked lymphoplasmacytic cells invasion under the dense fibrosis (Fig. 4b) was observed. These cells formed numerous lymphoid follicles containing germinal centers (Fig. 4c).

**Fig. 5:**
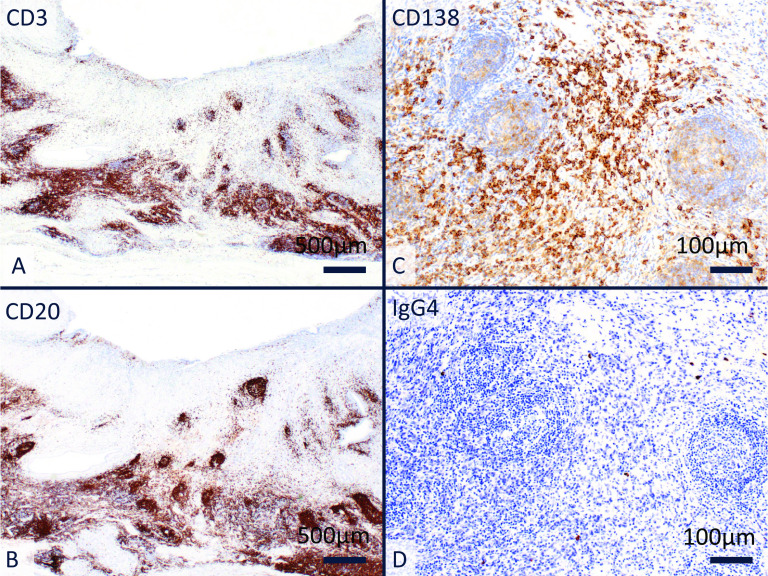
IHC. **A** The infiltrating lymphocytes constitute a heterogeneous population of cells that are positive for CD3. **B** Infiltrating cells also positive for CD20. **C** Many CD138-positive plasma cells are scattered around the lymphoid follicles. **D** There are few IgG4-positive plasma cells in this lesion

IHC showed positive for CD3 (Fig. 5a), CD20 (Fig. 5b) and CD79a in infiltrated lymphoplasmacytic cells under the thickening fibrosis. Although many CD138-positive plasma cells were scattered around the lymphoid follicles (Fig. 5c), IgG4-positive plasma cells were few (Fig. 5d). Based on these findings, this unique lesion was diagnosed as FC. The patient has been followed up as an outpatient, and she has not experienced recurrence at 42 months after surgery.

## Discussion and conclusions

Benign bile duct stenosis comprises various conditions, including primary sclerosing cholangitis (PSC), IgG4-related sclerosing cholangitis (IgG4-SC), post-traumatic bile duct stenosis, iatrogenic bile duct stenosis, etc. FC is an extremely rare disease that differs from those established benign bile duct disorders. Only 11 cases have been reported since the first report by Aoki et al. in 2003 [1]. According to previous reports (including our case), the median age was 61 years (42–77 years), FC appeared more prevalent among relatively young patients (Table 1). The male-to-female ratio was 5:7, with a slight female predominance. Two cases presented abdominal pain [2, 3] and 4 cases had jaundice-related symptoms [3, 4], while the other 6 cases were asymptomatic [1, 3, 5, 6]. Elevated hepatic enzymes were observed in all but one case. There were no cases of elevated serum CEA levels. Only two cases presented with elevated CA19-9 [3], this may be related to bile duct obstruction.

**Table 1:** Literature review of cases diagnosed with follicular cholangitis

Case	Ref. no.	Author	Year	Age	Sex	Symptoms	Increase of bilirubin, liver, bile duct enzyme	Tumor marker		Location	Preoperative diagnosis	PET/CT	T cell marker		B cell marker		IgG4(IHC)	Treatment	Prognosis
								CEA	CA19-9				CD3	CD4	CD20	CD79a			
1	[1]	Aoki	2003	57	F	None	+	−	−	Hilar	HC	ND	+	+	+	+	ND	Extended right hepatectomy	ND
2	[2]	Lee	2005	61	M	Pain	+	ND	ND	Hilar, right and left hepatic duct, cystic duct, gallbladder neck	HC	ND	ND	ND	ND	ND	ND	Extrahepatic bile duct and part of the right hepatic duct resection	ND
3	[5]	Fujita	2010	47	M	None	+	−	−	Hilar, right hepatic duct	HC	ND	+	+	+	+	−	Extended right hepatectomy	10 m alive
4	[5]	Fujita	2010	44	F	None	+	−	−	Hilar	HC	ND	+	+	+	+	−	Extended left hepatectomy	24 m dead
5	[3]	Zen	2012	73	F	Pain, jaundice	+	ND	+	Right hepatic duct	HC	ND	+	ND	+	ND	Few	Right hepatectomy	ND
6	[3]	Zen	2012	70	M	None	+	ND	+	Left perihilar duct	HC	ND	+	ND	+	ND	Few	Left hepatectomy	ND
7	[3]	Zen	2012	42	F	Jaundice, itching	+	ND	−	Hilar	PSC	ND	+	ND	+	ND	Few	Liver transplantation	ND
8	[9]	Fujii	2014	60	F	Itching, fatigue	+	−	−	Hilar, right and left hepatic duct	HC	ND	ND	ND	ND	ND	Few	Left trisegmentectomy	24 m alive
9	[7]	Saito	2016	69	F	Unknown	−	−	−	B3	Hepatolithiasis	ND	ND	+	+	ND	−	Left hepatectomy	12 m alive
10	[6]	Chang	2019	60	M	None	+	−	−	B5, 8	IHC	−	+	ND	+	ND	ND	Right hepatectomy and caudate lobectomy	18 m alive
11	[4]	Kosone	2020	70	M	Jaundice	+	−	−	Hilar	HC	+	ND	ND	ND	ND	Few	Hilar cholangiocarcinoma	30 m alive
12	Our case	Koneri	2023	77	F	None	+	−	−	Distal bile duct	Distal bile duct cancer	−	+	ND	+	+	−	Subtotal stomach-preserving pancreaticoduodenectomy	42 m alive

*ND* no data, + positive, − negative, *HC* hilar cholangiocarcinoma, *PSC* primary sclerosing cholangitis, *IHC* intrahepatic cholangiocarcinoma

Endoscopic retrograde cholangiopancreatography (ERCP), CE-CT and MRCP are performed in almost all cases. Localized bile duct stenosis and peripheral bile ducts dilatation have been reported in all cases. FC occurred predominantly in the intrahepatic or hilar bile ducts (Table 1), but our case is in the distal bile duct. Unfortunately, it is unclear why the lesions occur more frequently in the proximal bile ducts than in the distal biliary tract. Aoki et al. reported that “holly-like appearance” on ERCP was a characteristic imaging finding of this disease [1]; however, no characteristic findings that differentiate FC from other disease clearly have been reported. In our case, CE-CT did not reveal “holly-like appearance”, but a unique finding of “multiple overlapping leaf-like folds appearance”. This appearance will be helpful in diagnosing FC in the future. ^18^F-FDG-PET/CT was performed in only 3 cases. Although 2 cases did not reveal ^18^F-FDG uptake, Kosone et al. reported a case of significant FDG accumulation [4].

Regarding pathological findings, Saito et al. reported that FC shows dense fibrosis of bile duct under the mucosal layer with marked formation of lymph follicles containing germinal centers [7]. Those characteristic findings will provide clues for distinguishing FC from PSC or IgG4-SC. PSC often exhibits periductal fibrosis known as onion skin-like fibrosis and mucosal ulceration. IgG4-SC show obliterative phlebitis and storiform fibrosis [8]. In this case, there were no microscopic findings corresponding to PSC or IgG4-SC.

Previous articles indicate that IHC is also important in diagnosis. Some papers reported that FC show positive for both T cell (CD3, CD4) and B cell markers (CD20, CD79a) [5, 9]. On the other hand, negative for IgG4 (marker for IgG4-SC) and bcl-2 (follicular lymphoma). In addition, our case showed no evidence of phlebitis obliterans on Elastica van Gieson stain, negating IgG4-SC. Light chain restriction was not detected in κ and λ in situ hybridization, thus ruling out malignant lymphoma. In our case, both microscopic findings and IHC were consistent with the previously reported characteristics of FC.

Referring to the treatment of FC, Fujita et al. reported a case of significant response to steroid [5]. If lymphocytic infiltration of the bile ducts is the primary etiology of this disease, steroids may have the ability to alleviate FC. Ten of the 12 reported cases were diagnosed with cholangiocarcinoma preoperatively (Table 1). However, since only one death from FC has been reported [5], FC can be considered a non-life-threatening disease.

At present, it is difficult to diagnose this disease preoperatively, but the accumulation of FC cases can lead to accurate diagnosis and appropriate treatment in the future.

## Data Availability

All data generated or analyzed during this study are included in the published article.

## Abbreviations

FC: Follicular cholangitis
ALP: Alkaline phosphatase
γ-GTP: γ-Glutamyl transpeptidase
CEA: Carcinoembryonic antigen
CA19-9: Carbohydrate antigen 19-9
CE-CT: Contrast-enhanced computed tomography
MRI: Magnetic resonance imaging
^18^F-FDG-PET/CT: ^18^F-fluorodeoxyglucose positron emission tomography–computed tomography
IHC: Immunohistochemical staining
MRCP: Magnetic resonance cholangiopancreatography
ERCP: Endoscopic retrograde cholangiopancreatography
PSC: Primary sclerosing cholangitis
IgG4-SC: IgG4-related sclerosing cholangitis
